# Annexin A4 and A6 induce membrane curvature and constriction during cell membrane repair

**DOI:** 10.1038/s41467-017-01743-6

**Published:** 2017-11-20

**Authors:** Theresa Louise Boye, Kenji Maeda, Weria Pezeshkian, Stine Lauritzen Sønder, Swantje Christin Haeger, Volker Gerke, Adam Cohen Simonsen, Jesper Nylandsted

**Affiliations:** 1 Membrane Integrity Group, Unit for Cell Death and Metabolism, Danish Cancer Society Research Center, Strandboulevarden 49, 2100 Copenhagen, Denmark; 2 Department of Physics, Chemistry and Pharmacy, University of Southern Denmark, Campusvej 55, 5230 Odense M, Denmark; 3 Institute of Medical Biochemistry, Center for Molecular Biology of Inflammation, University of Münster, Von-Esmarch-Str. 56, 48149 Münster, Germany

## Abstract

Efficient cell membrane repair mechanisms are essential for maintaining membrane integrity and thus for cell life. Here we show that the Ca^2+^- and phospholipid-binding proteins annexin A4 and A6 are involved in plasma membrane repair and needed for rapid closure of micron-size holes. We demonstrate that annexin A4 binds to artificial membranes and generates curvature force initiated from free edges, whereas annexin A6 induces constriction force. In cells, plasma membrane injury and Ca^2+^ influx recruit annexin A4 to the vicinity of membrane wound edges where its homo-trimerization leads to membrane curvature near the edges. We propose that curvature force is utilized together with annexin A6-mediated constriction force to pull the wound edges together for eventual fusion. We show that annexin A4 can counteract various plasma membrane disruptions including holes of several micrometers indicating that induction of curvature force around wound edges is an early key event in cell membrane repair.

## Abstract (web summary)

The role of annexins in cell membrane repair is largely undefined. Here the authors use a model lipid bilayer to show that annexin A4 induces curvature at the membrane free edge and annexin A6 induces constriction force, and find that both annexins are recruited to wound edges in cells and are required for repair.

## Introduction

The plasma membrane repair system is essentially required to cope with membrane disruptions and thereby sustains cell life. Yet, the underlying molecular mechanisms used to repair membrane lesions in eukaryotic cells are not well characterized^1,2^. However, studies in different eukaryotic cell types reveal that the Ca^2+^-triggered repair system is shared with other cellular functions and involves cytoskeleton reorganization^3^, membrane internalization^4^, or shedding of damaged membrane^5^ involving both endo- and exocytosis mechanisms^6,7^.

Annexin A4 (ANXA4) belongs to the family of human annexin proteins (ANXA1–ANXA11 and ANXA13) whose function is only partially understood. ANXA4 protein stands out as one of the smallest annexin family members containing a short N-terminal region, whereas the largest member, ANXA6 is composed of two annexin cores. Annexins are activated by Ca^2+^ binding through their highly conserved C-terminal core domain enabling them to bind anionic phospholipids in plasma- and intracellular membranes^8^. Annexin family members, ANXA1 and ANXA2, were the first to be associated with plasma membrane repair in dysferlin-deficient muscular dystrophy and proposed to promote wound healing by fusing intracellular vesicles to the plasma membrane based on their ability to aggregate and fuse liposomes in vitro^9^. In addition, ANXA6 was recently reported to be required for repair of sarcolemma lesions in muscle cells where it forms a tight repair cap at the site of injury^10^. However, recent findings suggest that annexins, besides their membrane fusion capacities, also have more specific functions in the repair response. For example, ANXA5 is recruited to the vicinity of a membrane hole where it self-assembles into 2D-ordered protein arrays, which appear to restrict wound expansion during the repair process^11^. In line with this, ANXA4 can also self-assemble into trimers on membrane surfaces, which is thought to restrict the mobility of phospholipids and proteins in the membrane^12^. Annexin proteins appear to be instrumental for coping with abiotic stress responses in plants, and human annexins including ANXA4, are overexpressed in various cancer types characterized by enhanced intrinsic stress^13–15^. Hence, eukaryotic cells probably cope with membrane stress and injuries to their cell membrane by upregulating their arsenal of annexin proteins. In the light of these results, we hypothesized that ANXA4 can counteract plasma membrane stress by a cell membrane repair mechanism. Thus, we examined the function of ANXA4 on artificial membranes and in cells challenged to different stress conditions that trigger plasma membrane disruptions. Using a model lipid bilayer, we provide evidence that ANXA4 induces curvature at the membrane-free edge, whereas ANXA6 induces constriction force. Moreover, both annexins are recruited to wound edges in cells and are required for repair. We present a biophysical model showing that the combined effect of membrane curvature and constriction deliver force to contract the wound edge for eventual closure.

## Results

### ANXA4 repairs plasma membrane stress-induced lesions

**Fig. 1:**
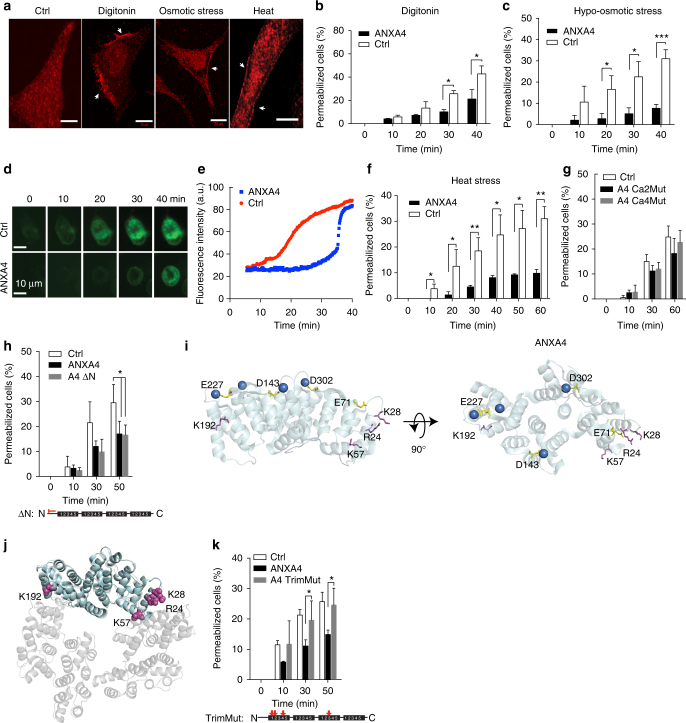
ANXA4 counteracts plasma membrane injuries triggered by detergent, hypo-osmotic stress, and heat shock. **a** Immunofluorescence images showing endogenous ANXA4 protein localization in HeLa cells (white arrows) exposed to digitonin (20 µg/ml, 15 min), hypo-osmotic stress (25 mOsm/l, 10 min) or 47 °C heat shock (5 min) (scale bar, 20 µm). Plasma membrane integrity assays in cells overexpressing ANXA4-RFP or Ctrl-RFP and **b** treated with digitonin (20 µg/ml) in HeLa cells, or **c** MCF7 cells exposed to hypo-osmotic stress (25 mOsm/l) and assayed with impermeable Hoechst-33258. Also, see Supplementary Fig. 1. **d** Images showing FM1-43 dye uptake in MCF7 cells exposed to hypo-osmotic stress and **e** corresponding intensity plot. **f** Plasma membrane integrity upon 55 °C heat shock in MCF7 cells expressing ANXA4 wild type, or **g** Ca^2+^-binding-deficient mutants, **h** mutant lacking N terminus (aa 4-13) (ΔN). **i** Residues mutated in the Ca2Mut/Ca4Mut and the TrimMut ANXA4 mutants are indicated with yellow and pink sticks, respectively, on the crystal structure of ANXA4 (PDB entry code: 2zoc) displayed in transparent light blue cartoon with bound calcium ions as blue spheres. **j** ANXA4 (light blue) is structurally aligned on a subunit of homotrimer observed in the crystal structure of ANXA5 (transparent gray, PDB entry code: 1a8a) and the residues mutated in the ANXA4- TrimMut mutant displayed as pink spheres. The two structures are aligned to a root-mean-square deviation (RMSD) value of 0.942 Å over 302 Cα atoms (PyMOL Molecular Graphics System, Version 1.7.6.4, Schrödinger, LLC). k Membrane integrity upon 55 °C heat shock in MCF7 cells expressing ANXA4-TrimMut as compared to wtANXA4 and control. Means of three independent experiments measuring >40 cells for each condition. Error bars represent S.D. for three independent experiments. *P* values based on *t*-test: *P ≤ 0.05, **P ≤ 0.01, ***P ≤ 0.001

To investigate if ANXA4 can counteract plasma membrane disruptions, human HeLa cervix carcinoma or MCF7 breast carcinoma cells were injured by exposing them to detergent, hypo-osmotic stress, or heat shock. These treatments triggered translocation of endogenous ANXA4 to the plasma membrane within 10–15 min as visualized in HeLa cells by immunofluorescence staining (Fig. 1a). HeLa cells overexpressing fluorescently tagged ANXA4 were wounded by the membrane pore-forming detergent digitonin and plasma membrane integrity was measured by impermeable Hoechst exclusion assay. ANXA4-RFP expression reduced the percentage of permeabilized cells significantly as compared to control in both Hela (Fig. 1b) and MCF7 cells (Supplementary Fig. 1a, c, e), whereas ANXA5 conferred only minor repair after 10 min (Supplementary Fig. 1b, d).

Next, MCF7 cells were exposed to hypo-osmotic stress, which swells cells and eventually induces lesions in the plasma membrane due to overstretching. Here ANXA4-RFP-expressing cells showed improved repair with four- to fivefold fewer permeabilized cells after 40 min (Fig. 1c) and ability to maintain plasma membrane integrity significantly longer as compared to Ctrl-RFP cells (Fig. 1d, e).

Lastly, MCF7 cells were exposed to 43 °C heat stress, which enhances thermal energy of membrane phospholipids and induces plasma membrane lesions due to the increased thermal motion and random rearrangement of lipids^16^. Here ANXA4-RFP-expressing cells coped with heat stress and maintained membrane integrity twice as long as Ctrl-RFP cells (Supplementary Fig. 1f, g). Moreover, ANXA4 expression improved cell membrane repair even after severe heat shock triggered by incubating cells in 55 °C pre-warmed medium while cells were kept in a 43 °C incubator (Fig. 1f).

To elucidate structural domains required for ANXA4-mediated repair, different mutants were expressed in MCF7 cells and tested for their repair ability upon 55 °C heat shock. Expression of ANXA4 Ca^2+^-binding-deficient mutants with two (Ca2Mut) or four (Ca4Mut) Ca^2+^-binding sites inactivated failed to repair indicating that Ca^2+^ activation is required for the repair function (Fig. 1g). Deletion of residues 4–13 of the N-terminal region (ΔN) did not compromise repair after heat shock, suggesting that the core domain of ANXA4 is sufficient for repair (Fig. 1h). To further characterize structural features, we mutated four positively charged residues in ANXA4, which are conserved in both ANXA4 and ANXA5 and were previously demonstrated to be required for the self-assembly of ANXA5 into trimers on membranes^11^ (Fig. 1i, j). ANXA4 trimer-deficient mutant (TrimMut) failed to repair upon heat shock, suggesting that ANXA4 trimer formation at the cell membrane is required for its repair ability (Fig. 1j, k). Next, we examined the behavior of ANXA4 wild type and mutants upon localized plasma membrane damage triggered by laser irradiation. HeLa cells overexpressing wild-type ANXA4-GFP and ANXA4 mutants fused to RFP were injured by laser and their translocation kinetics measured at the site of injury. Both Ca^2+^-binding-deficient mutants failed to translocate to the plasma membrane upon injury (Supplementary Fig. 1k, l), whereas the trimer-deficient TrimMut mutant accumulated similarly as wild-type ANXA4 (Supplementary Fig. 1m). Translocation of the ΔN mutant was delayed as compared to wild type, but the mutant was still recruited to the plasma membrane after damage (Supplementary Fig. 1n). Thus, our data indicate that Ca^2+^ binding is essential for the initial translocation of ANXA4 to plasma membrane but trimer formation and the N-terminal region are dispensable. Taken together, our results suggest that ANXA4 is involved in Ca^2+^-triggered plasma membrane repair and that trimer formation is important for this function.

### ANXA4 is required for repair of large cell membrane injuries

**Fig. 2:**
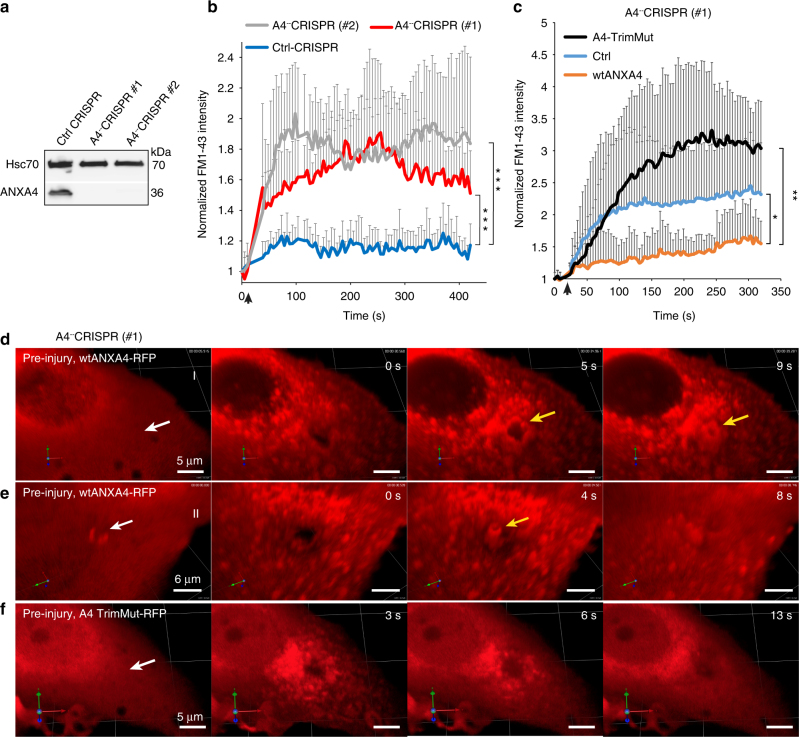
ANXA4 is needed for repair in MCF7 cells. **a** Immunoblot of lysates from MCF7A4^−^-CRISPR cells with disrupted ANXA4 gene expression. Hsc70 served as internal control for equal loading. **b** Plasma membrane repair kinetics upon laser injury measured by membrane impermeable FM1-43 dye influx in **b** CRISPR clones and in **c** MCF7A4^−^-CRISPR (#1) cells expressing wild-type ANXA4-RFP, ANXA4 TrimMut-RFP or Ctrl-RFP. Error bars represent S.D. for at least eight independent cells per condition. *P* values based on *t*-test: *P ≤ 0.05, **P ≤ 0.01. **d** Sequential 3D images of MCF7A4^--^-CRISPR (#1) with reintroduced ANXA4-RFP and exposed to a large injury by shooting through the cell by laser (injury site marked by white arrow). ANXA4-RFP translocation to wound edges and constriction is marked by yellow arrow (also, see Supplementary Movie 1). **e** Example of ANXA4-RFP-induced curvature and funnel formation (yellow arrow) created from the bottom of the plasma membrane after laser injury. **f** Trimer-deficient ANXA4-RFP mutant (A4-TrimMut) failed to induce curvature upon laser injury (see also Supplementary Movie 1)

To address if ANXA4 is required for repair, we generated MCF7 clones with CRISPR/cas9- disrupted ANXA4 gene expression (MCF7A4^−^-CRISPR) (Fig. 2a). First, MCF7A4^−^-CRISPR clones were injured by laser and their membrane repair kinetics compared by measuring entry of impermeable FM1-43 dye into the cytoplasm. Interestingly, MCF7A4^−^-CRISPR clones were compromised in their repair as compared to control, suggesting that these cells depend on ANXA4 for efficient wound healing (Fig. 2b). To gain insight into the repair mechanism, we introduced wild-type ANXA4-RFP, trimer-deficient mutant TrimMut-RFP, or Ctrl-RFP into MCF7A4^−^CRISPR cells and analyzed repair kinetics after laser injury by FM1-43 dye exclusion assay. Here ANXA4 improved repair compared to control, whereas A4-TrimMut was worsening repair as compared to both wild-type ANXA4 and Ctrl (Fig. 2c). Moreover, reintroduction of wild-type ANXA4 into MCF7A4^−^CRISPR cells also reconstituted repair after 55 °C heat shock as compared to Ctrl (Supplementary Fig. 1o). These data suggest that ANXA4 is required for repair and that the trimer-deficient ANXA4 mutant has a dominant-negative effect on the repair machinery at the wound site (Fig. 2c).

Next, MCF7A4^−^CRISPR cells were injured by shooting a hole through the cell, which generates wounds of micron size with visible edges (Supplementary Fig. 2a). Subsequent imaging of wild-type ANXA4- or Ctrl-RFP behavior during wound healing revealed that ANXA4-RFP localized to the edges and appeared to induce wound closure within 10 s in contrast to Ctrl (Supplementary Fig. 2a). To further characterize this phenomenon, we injured MCF7A4^−^CRISPR cells by laser and imaged cells in 3D (Fig. 2d, e). Here, ANXA4-RFP was quickly recruited to wound edges and appeared to trigger an inward-directed membrane movement resulting in contraction of the membrane edges within 10 s (Fig. 2d and Supplementary Movie 1). Furthermore, in some cases, we observed a transient funnel-shaped structure originating from the bottom membrane in some cells before wound closure, which appeared in the center of the hole decorated by ANXA4-RFP protein (Fig. 2e). The trimer-deficient ANXA4 mutant failed to induce any funnel structure or contraction of wound edges suggesting that ANXA4 trimer formation is required for this function (Fig. 2f and Supplementary Movie 1). These observations suggest that ANXA4 can potentially manipulate the shape of membrane edges and that this function is important for wound healing. We tested if this effect involved spatial and timely actin accumulation around the wound using LifeAct-GFP as F-actin marker. Membrane injury triggered LifeAct-GFP accumulation 90 s later than ANXA4-RFP, indicating that actin is probably not responsible for the initial shaping and contraction of the wound edges (Supplementary Fig. 2b).

### ANXA4 induces curvature and roll-up of supported membranes

**Fig. 3:**
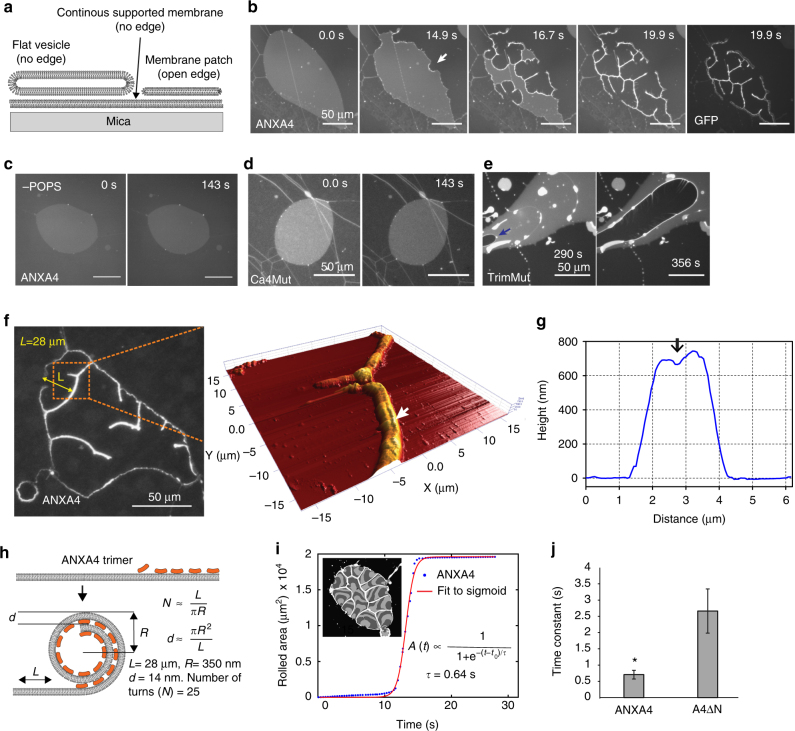
ANXA4 induces membrane curvature in supported membranes. **a** Schematic of supported membrane model composed of primary and secondary membranes. Non-vesicular membrane patches with open edges were used in the subsequent experiments (POPC/POPS, 9:1 molar ratio). **b** Sequential images before and after addition of ANXA4-GFP protein to a membrane patch stained with DiD in the presence of Ca^2+^ (last image GFP channel) (also, see Supplementary Movie 2). **c** Similar experiment as in **b** but without POPS in the supported membrane. **d**, **e** Mutants of ANXA4 proteins including **d** Ca4Mut or **e** TrimMut. Note: TrimMut exhibits >10 times slower rolling activity. **f** Fluorescence and matching AFM image of membrane patch after completed rolling induced by ANXA4 and **g** AFM-topography graph of two colliding membrane rolls. **h** Schematic of ANXA4-induced membrane rolling including a numeric estimate of the number of turns *N* and layer spacing *d*. **i** Calculation of time constant (*τ*) for membrane rolling and corresponding data for ANXA4. ANXA4-GFP shows similar rolling activity (data not shown). **j** Rolling time constants for ANXA4 and A4 ΔN, respectively. Error bars represent S.D. for three–five independent experiments. *P* values based on *t*-test: *P ≤ 0.05, ***P ≤ 0.001

To explore whether ANXA4 can influence the conformation of membranes, we tested the impact of ANXA4 on the morphology of artificial membranes. First, we measured the binding of recombinant ANXA4-GFP protein to giant liposomes composed of phosphatidylcholine (DOPC) and anionic phospholipids. ANXA4 localized to the surface of liposomes containing negatively charged phosphatidylserine (DOPS) or phosphatidic acid (DOPA) with highest binding to DOPA (Supplementary Fig. 3a). However, by this approach, we were not able to detect changes in membrane topography upon ANXA4 binding. Thus, we turned to a solid-supported membrane model composed of both flat vesicles and membrane patches laying on a primary membrane formed after hydration of a precursor lipid film^17^ (Fig. 3a). Here membrane patches resemble freestanding membranes and contain stable- free edges relevant for studying membrane conformation of a hole in response to annexins^18^. Interestingly, addition and binding of recombinant ANXA4-GFP protein to membranes induced extensive curvature initiating from the edges, which resulted in apparent rolling of entire membrane patches within 5 s (Fig. 3b; Supplementary Fig. 3b; and Supplementary Movie 2, left panel). In the absence of phosphatidylserine in membranes (Fig. 3c), absence of Ca^2+^ (Supplementary Fig. 3c), or with mutations in Ca^2+^-binding sites of ANXA4 (Ca4Mut) (Fig. 3d), this capability was compromised. To characterize this phenomenon further, we incubated supported membranes with ANXA4-TrimMut protein deficient in trimer formation. Interestingly, the TrimMut mutant delayed rolling onset and slowed rolling speed by tenfold (Fig. 3e and Supplementary Movie 2, right panel).

We assessed membrane topography after ANXA4-induced rolling by atomic force microscopy (AFM), which revealed a groove in situations where two rolls had collided from opposite sides, thus supporting the rolling mechanism (Fig. 3f, g). The number of turns in a roll was estimated to 25 and the repeat distance to 14 nm by considering the height of rolls and rolling length in direction perpendicular to the rolling axis of a specific patch region (Fig. 3h and Supplementary Fig. 3d). Further, by evaluating kinetics in the decrease in membrane patch area upon annexin addition (Fig. 3i), we determined a characteristic time constant for membrane rolling, which revealed faster rolling for wild-type ANXA4 than the ΔN mutant lacking the N terminus (Fig. 3j). Here, Ca4Mut and TrimMut mutants that compromised rolling did not follow this kinetic model. In summary, these results show that ANXA4 directly induces membrane curvature leading to rolling starting from free membrane edges, which is dependent on trimer formation and Ca^2+^ activation.

### Membrane curvature at wound edges is energetically favored

**Fig. 4:**
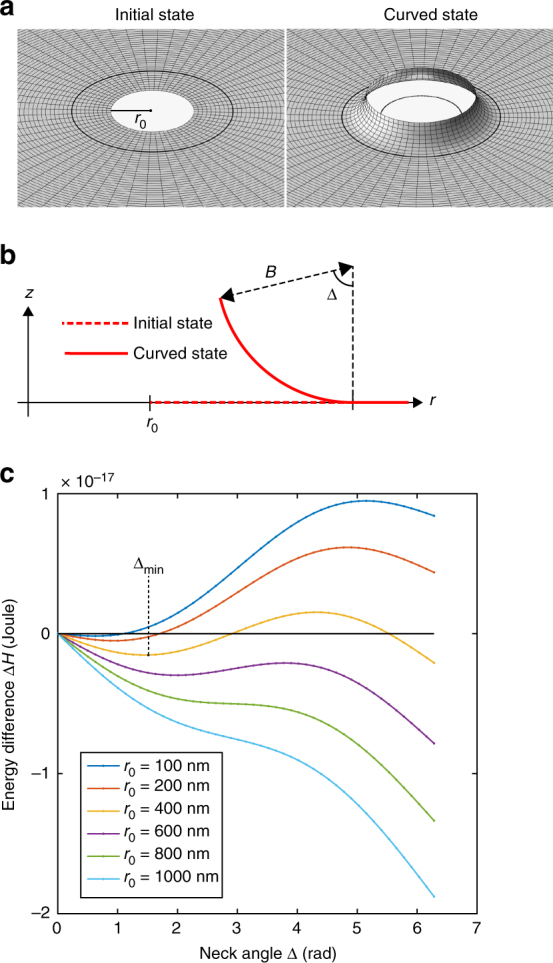
Biophysical model for ANXA4-induced curvature during repair. **a** Initial and curved state of a circular membrane hole. The solid area inside the solid circle is the region contributing to the change in curvature elastic energy between the initial and curved states. **b** Profile of the neck and definitions: initial hole radius *r*_0_, the neck angle Δ and curvature radius *B*. **c** Energy difference Δ*H* as a function of Δ for varying values of the initial hole size. When *r*_0_ is below a critical hole radius *r*_0_* there is a minimum in Δ*H* corresponding to a energetically favored neck angle Δmin. In the plot *r*_0_ ≈ 800 nm. For *r*_0_ > *r*_0_*, Δ*H* decreases monotonically with Δ meaning that continuous rolling is favored (also, see Supplementary Notes 1 and 2 for detailed description)

To characterize the potential role of curvature in plasma membrane wound healing, we established a theoretical model to estimate the energetics of curvature formation. Here, an initially planar membrane with a hole of radius *r*_0_ is transformed into a neck geometry with a circular neck profile upon ANXA4 binding (Fig. 4a). The degree of neck formation is characterized by the neck angle Δ defined as Δ = 0 corresponding to no curvature and Δ = 2*π* to one complete roll (Fig. 4b and Supplementary Note 1). The energy difference between the initial and curved state is based on contributions from (1) the membrane curvature elastic energy and (2) the line tension at the hole edge. Thus, the effect of ANXA4 binding is modeled as a spontaneous curvature term *c*_0_ in the Helfrich elastic energy expression^19^. Accordingly, the initial planar membrane will be subject to a curvature stress upon ANXA4 binding, which is lowered by bending. The numerical results (Fig. 4c) show that the neck formation is energetically unfavorable for small initial hole sizes (*r*_0_≪100 nm) because of the relative importance of line tension, which dominates over curvature elastic energy. As the initial hole radius increases, an energy minimum develops corresponding to formation of a neck with a finite neck angle Δ_min_. For larger holes exceeding a critical radius, the minimum disappears and continuous rolling of the membrane edge is favored (Δ > 2*π*) (Fig. 4c. See, also Supplementary Notes 1 and 2 for details). The result in this limit agrees with our experimental observation of rolling in membrane patches where the membrane edge is approximately linear and equivalent to that of an infinitely large hole (Fig. 3). Taken together, our data and model suggest that ANXA4-triggered out-of-plane curvature of wound edges is energetically favored, which is important in the initial phase of repair by providing mechanical curvature force during wound closure.

### Annexin A6 induces constriction of supported membranes

**Fig. 5:**
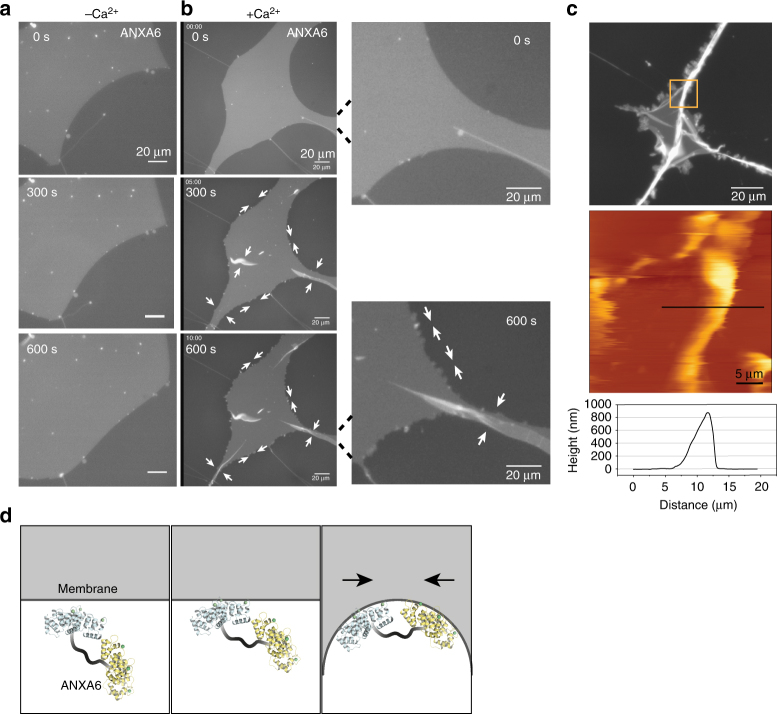
ANXA6 triggers in-plane constriction of membrane edges and shrinkage of supported membrane islands. **a** Sequential images before and after addition of ANXA6 protein to a membrane patch stained with DiD in the absence of Ca^2+^ or **b** in the presence of Ca^2+^. Note that ANXA6 induces in-plane folding of membrane edges (lower panel, magnified image) as well as larger out-of-plane folds (white arrows). **c** Fluorescence and matching AFM images of membrane patch after completed ANXA6 folding. Lower panel: corresponding AFM-topography graph. **d** Model for ANXA6-triggered local membrane constriction by cross bridging of adjacent membrane via the two core domains

ANXA4 curvature at membrane edges could potentially expand a wound hole further unless the edges are stabilized and/or the force is orientated toward resealing by, e.g., another annexin(s). Annexin A6 (ANXA6) has an unusual structure composed of two annexin cores that may bridge two adjacent membranes and was previously reported to be implicated in plasma membrane repair^10,20^. Thus, we tested the impact of ANXA6 protein on supported membranes either in the absence or presence of Ca^2+^ (Fig. 5a, b). Interestingly, ANXA6 triggered an in-plane folding of the membrane edges in a Ca^2+^-dependent manner (Fig. 5b), which resulted in gradual shrinkage of the whole membrane island. The folding phenotype, which was distinctly different from ANXA4-induced rolling and appeared at the edges as small meanderings and then continued to larger areas resulting in folds with a height of up to one micron (Fig. 5c). These data suggest that ANXA6 binding induces contraction of membrane possibly due to its cross-bridging ability, which could play a role during wound healing by constricting wound edges (Fig. 5d).

### ANXA4 and ANXA6 are required for repair at wound edges

**Fig. 6:**
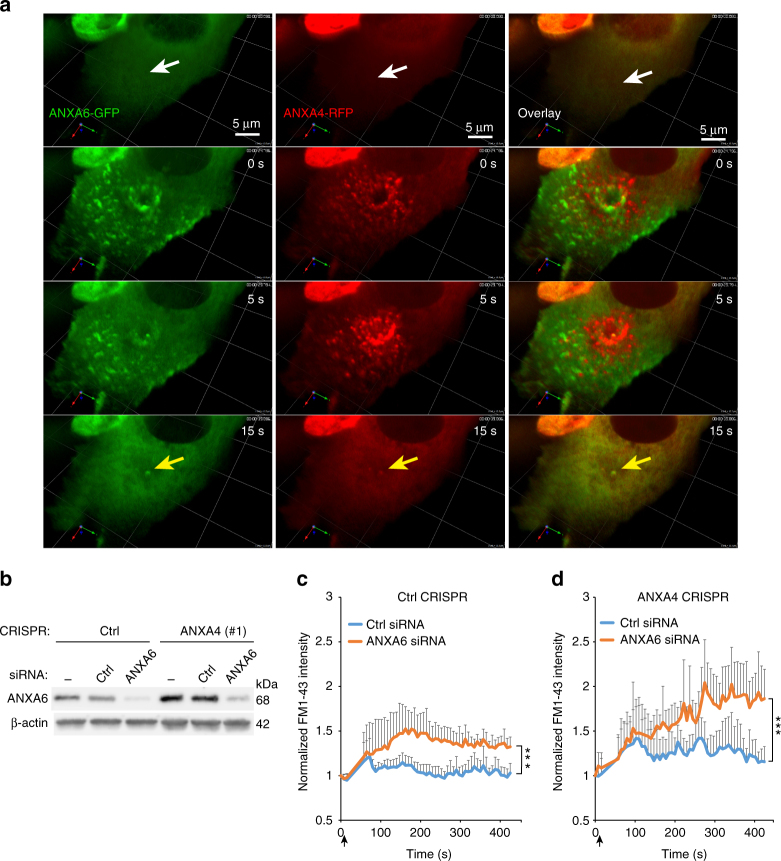
ANXA6 is recruited to wound edges and required for repair. **a** Representative sequential 3D images of MCF cell showing the localization of ANXA6-GFP and ANXA4-RFP in response to laser injury (white arrows indicate injury site). Cells were injured by shooting through the cell to obtain a hole with clear edge. A putative repair cap defining where the edges have fused is visible in the middle (yellow arrow). **b** Immunoblot showing ANXA6 protein levels in CRISP Ctrl and MCF7A4-CRISPR (#1) cells or in similar cells treated with ANXA6 or Ctrl siRNA for 48 h. **c** Cell membrane repair kinetics in ANXA6 siRNA-treated cells compared to Ctrl siRNA-treated cells upon laser injury measured by membrane impermeable FM1-43 dye influx in MCF7Ctrl CRISPR or **d** MCF7A4^--^CRISPR (#1). Error bars represent S.D. for at least seven independent cells per condition. *P* values based on *t*-test: *P ≤ 0.05, **P ≤ 0.01. **P ≤ 0.001

To investigate the role of ANXA6 in wound healing, we imaged the behavior of ANXA4-RFP and ANXA6-GFP in MCF7 cells exposed to large injuries by laser (wound diameter: 1–3 µm). Here, ANXA6-GFP appeared at the wound site a few seconds before ANXA4-RFP followed by co-localization, -contraction of wound edges, and closure and subsequent diffusion of both proteins back to the cytoplasm within 15 s (Fig. 6a). A putative repair cap appeared in the middle of the hole after repair containing both ANXA4-RFP and ANXA6-GFP, which might indicate the point where membranes had fused (Fig. 6a, yellow arrow). Intriguingly, sometimes we observed that cells upon laser injury-initiated ANXA4/ANXA6 translocation followed by wound closure, but then employed a second repair strategy involving excision of the whole part of their damaged membrane (Supplementary Fig. 4b and Supplementary Movie 3). These results show that ANXA4/ANXA6 recruitment serves as the initial repair response and that excision, which we have shown previously depends on S100A11/ANXA2 complex and actin polymerization, is the second-line repair response^21^.

**Fig. 7:**
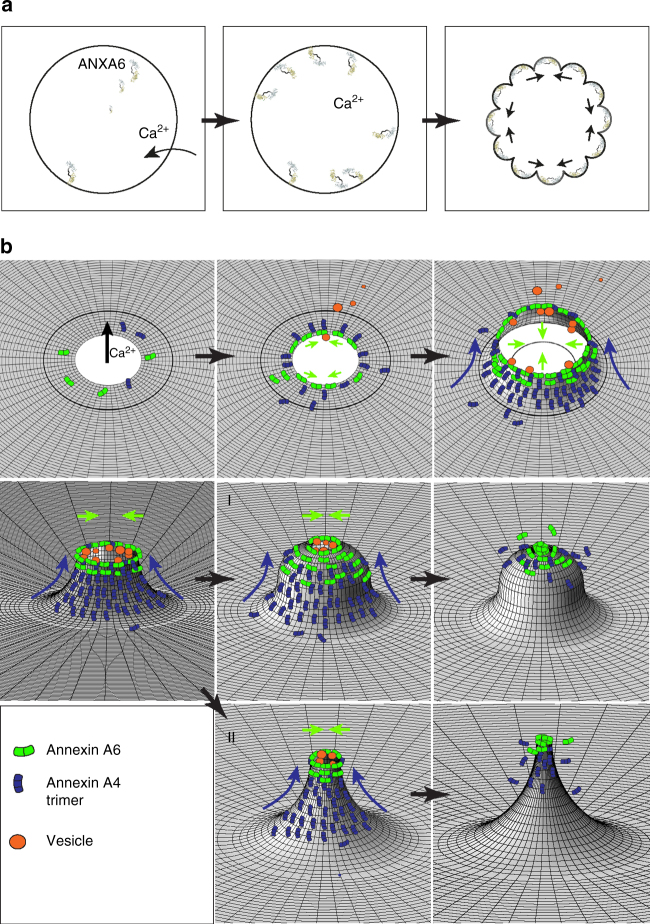
Proposed model for plasma membrane repair initiated by ANXA4 and ANXA6. **a** Binding of ANXA6 to wound edges induces local in-plane folding, which contracts the hole edges. **b** In uninjured cells, ANXA6 and ANXA4 are distributed uniformly as monomers in the cytoplasm. Upon local plasma membrane injury, Ca^2+^ influx results in recruitment of ANXA6 and ANXA4 to the membrane wound edges. ANXA6 initiates constriction of hole edges and may also cross-bridge patch vesicles translocated to the injury site. ANXA4 self-assembles into trimers that induce local out-of-plane curvature. The combined forces of constriction and curvature accelerate wound closure eventually leading to fusion of membrane edges. Vesicles recruited and fused to the wound edges contribute to repair by reducing wound size. The curvature structure triggered by ANXA4 can take several shapes including **I** round or **II** funnel-shaped or a more flat structure (not shown). Subsequent repair steps include local actin polymerization to fully restore the membrane after injury

To test whether ANXA6 is required for repair, we first compared ANXA6 protein levels in MCF7Ctrl-CRISPR or A4^−^CRISPR cells. Notably, MCF7A4^−^CRISPR cells appeared to compensate from lack of ANXA4 by upregulating their ANXA6 protein expression, suggesting that MCF7 cancer cells require at least one of these proteins to counteract plasma membrane damage (Fig. 6b). CRISPR clones were then depleted for ANXA6 by siRNA (to less than 10%) (Fig. 6b) and their repair kinetics were analyzed by monitoring the entry of membrane impermeable FM1-43 dye into cells following a laser-induced local injury to the cell membrane. Depletion of ANXA6 in Ctrl-CRISPR cells compromised repair efficacy as compared to Ctrl siRNA (Fig. 6c). However, siRNA knockdown of ANXA6 in MCF7A4^−^CRISPR cells compromised repair further, which resulted in enhanced sensitivity to laser injury and increased cell death (Fig. 6d and Supplementary Fig. 4a) indicating a need for both ANXA4 and ANXA6 for efficient repair. Thus, our data suggest that ANXA4 and ANXA6 are recruited to wound edges where they play distinct roles by providing curvature and contraction/stabilization, respectively, which provide the force to close a hole (Fig. 7).

## Discussion

Taken together, our results suggest that the annexin family members ANXA4 and ANXA6 besides their ability to aggregate membranes play specific roles in membrane repair. The remarkable wound healing efficacy in MCF7 cells enables cells to close holes of micron size within 20 s, which appears to occur by biophysical manipulation of wound edges organized by ANXA4 and ANXA6. The initial repair response happens within the time frame of Ca^2+^-triggered annexin translocation to the cell membrane and, at this point does not appear to involve actin polymerization. Plasma membrane repair mechanisms involve internalization via endocytosis, or exocytosis as observed from mechanical wounding or exposure to plasma membrane pore-forming agents^4,22,23^. However, endocytosis is likely to be limited to small lesions as the hole would have to be accommodated by the nascent endocytic compartment^24^. Also, exocytosis would probably be limited to be effective only for healing very small wounds. Alternatively, large wounds could be stopped through plugging by tight cross-linking of membranous organelles by Ca^2+^-triggered annexins^24,25^. Although it is possible that vesicles are fusing to wound edges, we did not observe any potential plug by imaging. However, from 4D imaging, it was evident that the wound closing mechanism involved contraction of wound edges within 10–15 s involving ANXA4 and ANXA6. Our previous results from invasive ErbB2-MCF7 show that actin polymerization is required at later stages to excise damaged membrane parts and restore membrane integrity and this process is orchestrated by the ANXA2/S100A11 complex^21^. To this end, we observed that repair of large wounds in MCF7 cells can involve both mechanism: first, recruitment of ANXA4/ANXA6 to the wound edges followed by hole contraction and next, excision of the damaged part of the plasma membrane (Supplementary Fig. 4b). This emphasizes the diversity of mechanisms used for plasma membrane repair. Thus, the repair strategy chosen by cells appears to be dependent on the extent of damage, spatial position of the injury, and cell type. Based on our results, we propose a model that depicts the various steps of initial repair response involving ANXA4 and ANXA6 (Fig. 7b). In resting cells, ANXA4 and ANXA6 are predominantly cytosolic. Following plasma membrane injury and Ca^2+^ influx, ANXA6 is recruited to the wound edges, where it initiates in-plane constriction of hole edges (Fig. 7a). Translocation of ANXA6 and other annexins to the wound enables aggregation and fusion of endosomes and other vesicles to the damaged membrane (Fig. 7b). Recruitment of monomeric ANXA4 to the injured membrane and self-association into trimers induce out-of-plane curvature of wound edges (Fig. 7b). The combination of ANXA6-induced constriction force and out-of-plane bending by ANXA4 direct the force toward wound closure. Other annexin family members are likely involved in the repair response by, e.g., fusing membranes including ANXA1, which is recruited directly to the injury site^21^. Furthermore, ANXA5 was recently reported involved in membrane repair by restricting wound expansion, which might collaborate with ANXA4- and ANXA6-induced forces^11^. To this end, we have identified a total of eight annexin family members in MCF7 cells, which are recruited to the cell membrane upon injury suggesting that more annexins take part in the repair network (our unpublished data).

Thus, ANXA4 and ANXA6 improve cells' ability to cope with stress-induced plasma membrane disruptions, which appears to be a strategy used by cancer cells to counteract frequent membrane injuries. Our results suggest that ANXA4 and ANXA6 play distinct roles in the plasma membrane repair response enabling cells to quickly cope with life-threatening membrane lesions.

## Methods

### Cell culture and treatments

HeLa (ATCC no.: CCL-2) and MCF7 (ATCC no.: HTB-22) cells originate from cervix carcinoma and human breast carcinoma, respectively, and were maintained in 6% fetal calf serum with antibiotics in a 37 °C incubator.

### Plasmid constructs and recombinant proteins

Expression plasmids containing human ANXA4, ANXA5, and ANXA6 with turbo-GFP/turboRFP C-terminal tag were purchased from OriGene Technologies. ANXA4 mutants deficient in Ca^2+^ binding were generated by PCR-based site-directed mutagenesis by replacing amino acid 71E, 143E (Ca2Mut), or furthermore 227E and 302D (Ca4Mut) in each annexin repeat to A^26^. To generate ANXA4 mutant deficient in trimer formation ANXA5 and ANXA4 alignment was performed to mutate similar amino acids required to inhibit ANXA5 trimer formation (24R, 28-K, 57-K and 192-K to E) (TrimerMut) based on crystal structure evidence^16^. In addition, an ANXA4 deletion mutant lacking amino acids 4–13 of the N-terminal region was generated (ΔN). ANXA4 mutants were generated in the mammalian pCMV6-AC-mRFP vector to express the proteins with C-terminally tagged red fluorescent protein (RFP). For protein production, ANXA4 and ANXA6 cDNA constructs were subcloned into the bacterial pEX-C-His vector (OriGene). Recombinant proteins were produced using immobilized metal-affinity chromatography followed by fast protein liquid chromatography. For CRISPR/Cas9-targeted ANXA4 gene disruption, the following target sequences were used: 5′-GCTTCAGGATTCAATGCCATGG-′3 (#1) and 5′-CCGATGAAGACGCCATTATTAG-′3 (#2). MCF7 cells transfected with all-in-one CRISPR/Cas9 plasmid (Sigma-Aldrich) were cell-sorted for GFP expression to generate single cell clones, which were sequenced to verify disruption of reading frame and analyzed for protein expression. siRNAs: ANXA6: 5′-CCUAUUGUGAUGCCAAAGA[dT][dT]-′3 (Sigma-Aldrich). AllStar negative control siRNA (Qiagen) was used as a negative control.

### Immunoblot analysis and immunocytochemistry

Protein lysates were separated by SDS–polyacrylamide gel electrophoresis and transferred to nitrocellulose membranes. Primary antibodies raised against human Annexin A4 (R&D Systems, Cat. No.: MAB4146. Dilution 1:500), Annexin A6 (Abcam, Cat. No.: ab31026. Dilution: 1:1000), heat shock cognate 70 kDa protein (Hsc70; N69. Dilution: 1:5000. Kindly provided by Boris Margulis, Russian Academy of Sciences, St Petersburg, Russia), tGFP/tRFP (OriGene, Cat. No.: TA150041/TA150061. Dilution 1:1000), heat shock protein 90 (Hsp90: BD Transduction Laboratories, Cat. No.: 610418. Dilution: 1:4000), and β-actin (Sigma-Aldrich, Cat. No.: A2228. Dilution 1:5000) were used followed by appropriate peroxidase-conjugated secondary antibodies (DAKO). Immunocytochemistry: Cells on coverslips were fixed in methanol and stained with indicated primary antibodies (1:100 dilution) including Annexin A4 (R&D Systems). Samples were incubated with the appropriate Alexa Fluor-594-coupled secondary antibodies (1:1000 dilution) (Molecular Probes) and images taken by a Zeiss confocal microscope.

### Membrane wounding experiments

Laser injury: cells were injured by selecting a circular region (diameter, 2 µm) and irradiating with a 355 nm UV ablation laser at 1.9% power setting, repetition rate 200 Hz, pulse energy >60 µJ, pulse length <4 ns (Rapp OptoElectronic), injury response was imaged with ×63 objective using a Nicon confocal microscope equipped with a PerkinElmer spinning disk. Cells were imaged every 4–10 s starting before injury and continuing for 3–8 min following injury. Volocity software (PerkinElmer) was used to control the hardware and to measure the kinetics of repair by monitoring uptake of impermeable FM1-43 dye as change in fluorescence intensity during the course of imaging. For detergent-induced PM injury, cells were incubated with 20 µg/ml digitonin (Sigma-Aldrich) or with varying digitonin concentrations. Hypo-osmotic-induced injury was performed by diluting growth media with H_2_O to achieve an osmolality of 25 mOsm/l. For heat-induced injury, cells were incubated in either 43 or 55 °C medium in a 43 °C microscope incubator. To measure membrane integrity and cytometry, cells were incubated with impermeable Hoechst-33258 (0.2 µg/ml) and FM1-43 dye (2 µM) and dye uptake imaged by a Carl Zeiss Axiovert 200M fluorescence time-lapse microscope and analyzed using MetaMorph software and ImageJ.

### Giant liposome formation

One percent agarose with ultralow melting temperature (Type I-A, Sigma-Aldrich) was used to generate the supporting agarose film for liposome formation. Lipid mixtures consisted of 89.4% phosphatidylcholine (DOPC; 1,2-di-(9Z-octadecenoyl)-sn-glycero-3-phosphocholine), 0.5% phosphatidylethanolamine bonded to polyethylene glycol (PEG), 0.1% Atto 647N lipid (PE-Atto 647N), and 10% signaling lipids; respectively, phosphatidic acid (DOPA; (1,2-di-(9Z-octadecenoyl)-sn-glycero-3-phosphate) and phosphatidylserine (DOPS; 1,2-di-(9Z-octadecenoyl)-sn-glycero-3-phospho-L-serine) (Avanti Polar Lipids). ANXA4-GFP protein binding to liposomes was assayed either in the absence or presence of 2 mM CaCl_2_.

### Supported membrane experiments

Mica substrates (Plano GmbH) were prepared from cleaved sheets and glued to glass coverslips using the silicone elastomer MED-6215 (Nusil Technology). Dry spin-coated lipid films of phosphatidylcholine (POPC; 1-hexadecanoyl-2–(9Z-octadecenoyl)-sn-glycero-3-phosphocholine) and phosphatidylserine (POPS; 1-hexadecanoyl-2–(9Z-octadecenoyl)-sn-glycero-3-phospho-L-serine) on mica substrates were prepared from a stock solution containing 10 mM total lipid (POPC, POPS, 9:1 molar ratio) and 0.5% DiD probe in methanol. A 40 µl droplet of the lipid stock was applied to the mica, spun on a spincoater and placed under vacuum in a desiccator for 10–12 h to ensure evaporation of the solvent. The spin-coated lipid film was hydrated in 50 mM TRIS buffer (2-Amino-2-(hydroxymethyl)propane-1,3-diol), 100 mM NaCl, 2 mM CaCl2, pH = 7.4 at 55 °C for 2 h. Then the sample was gently flushed with 55 °C buffer and buffer exchanged >10 times to prepare defined secondary bilayer patches resting on a continuous primary membrane. The response of bilayer patches to addition of annexin was monitored at 22 °C with time-lapse epi-fluorescence microscopy using a Nikon TE2000 inverted microscope (×40 objective, Nikon ELWD, Plan Fluor, NA = 0.6) and DiD-excitation with a Xenon lamp (Polychrome V, Till Photonics) at 640 nm and recorded with an EMCCD camera (Sensicam EM, PCO) operated with TILvision software (Till Photonics). Annexin in an absolute amount of 100 pmol was added to the fluid cell and the sample imaged at 3–10 fps depending on the response speed. GFP-labeled annexins were imaged simultaneously to DiD using a dual wavelength filtercube. Time-lapse sequences of membrane rolling were analyzed with methods written in MATLAB (The Mathworks). Briefly, the incremental rolled membrane area was determined by subtraction of subsequent frames in the sequence followed by binarization with a cutoff. The total rolled area as function of time was fitted by a logistic (sigmoid) function with a time constant *τ*, which provides a characteristic time scale for the rolling process.

### Data availability

All relevant data are available from the authors.

## Electronic supplementary material

Supplementary Information

Description of Additional Supplementary Files

Supplementary Movie 1

Supplementary Movie 2

Supplementary Movie 3
