# Supplementary material for: Annexin A4 and A6 induce membrane curvature and constriction during cell membrane repair
Source: Nat Commun. 2017 Nov 20;8:1623. doi: 10.1038/s41467-017-01743-6 (PMC5696365; doi:10.1038/s41467-017-01743-6)
Supplement: Supplementary file 1 — Supplementary Information [file 41467_2017_1743_MOESM1_ESM.pdf]

Supplementary Information

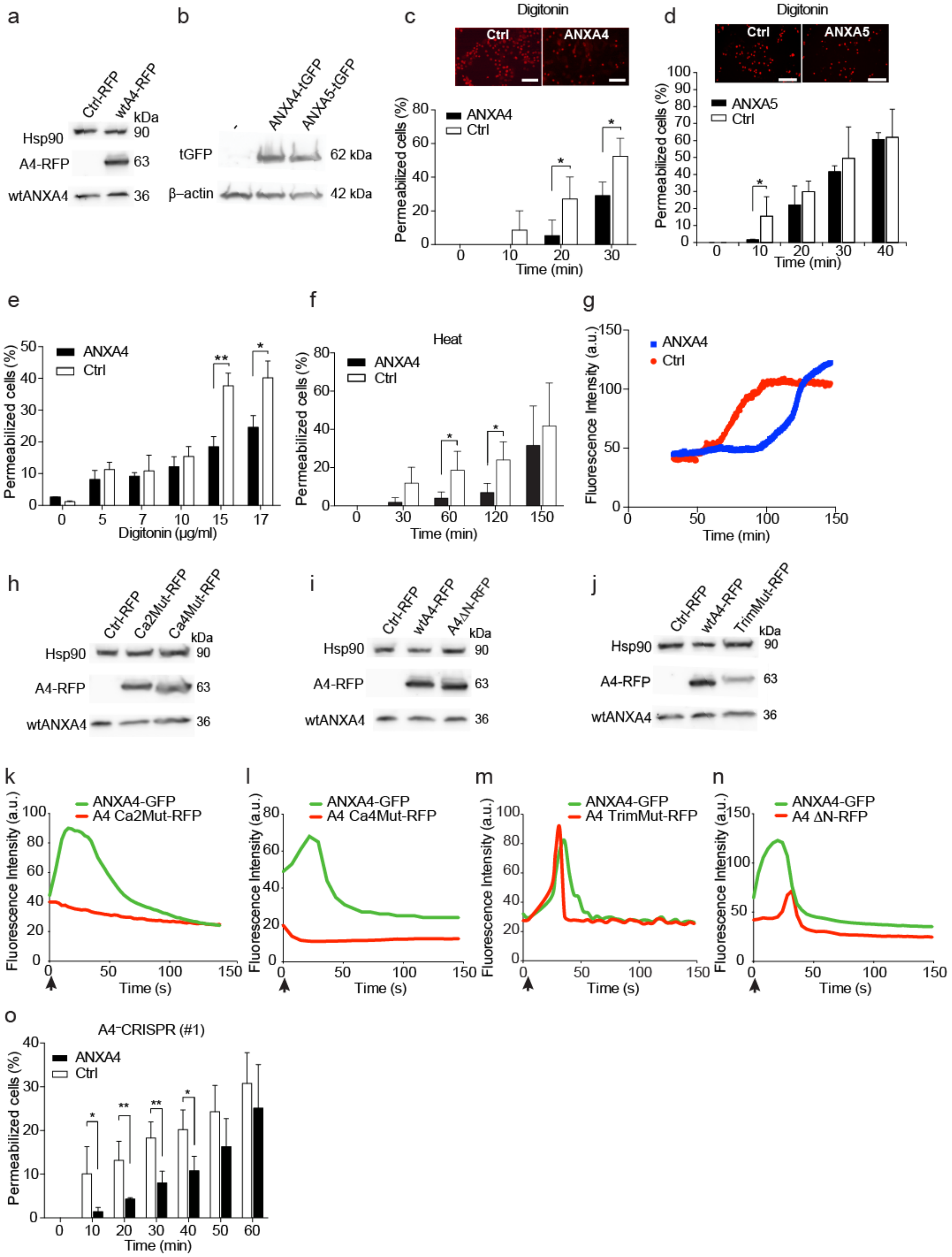

Supplementary Fig. 1 ANXA4 repairs injuries triggered by digitonin and heat shock treatment. **a** Immunoblot of lysates from MCF7 cells overexpressing wtANXA4 fused to tRFP or **b** to tGFP or ANXA5 fused to tGFP. **c**

Plasma membrane integrity after treatment with digitonin (20 µg/mL) in MCF7 cells overexpressing ANXA4-RFP or **d** ANXA5-GFP as compared to Ctrl-GFP expressing cells. Permeabilized cells were imaged by live-cell microscopy using impermeable Hoechst-33258 influx as readout. Upper panel: representative images extracted from time-lapse movies showing permeabilized Hoechst-33258 positive nuclei (red pseudo-color). **e** MCF7 cells treated with increasing digitonin concentration and plasma membrane integrity measured by propidium iodide exclusion assay. **f** Quantification of 43°C heat shock induced permeabilization of MCF7 cells expressing ANXA4-RFP or Ctrl-RFP as measured by Hoechst-33258 influx. **g** Corresponding plasma membrane repair kinetics measured by FM1-43 uptake in cells exposed to 43°C heat shock in two representative cells. Quantification of permeabilized cells was performed from ≥3 independent experiments for each condition measuring >40 cells in each experiment. P-values based on t-test: $^*P \leq 0.05$, $^{**}P \leq 0.01$, $^{***}P \leq 0.001$. **h** Immunoblot of lysates from MCF7 cells overexpressing Ca2Mut-RFP or Ca4Mut-RFP, **i** A4 ΔN-RFP or **j** TrimMut-RFP. **k-n** Translocation kinetics of wildtype ANXA4-GFP and different mutants upon laser injury in HeLa cells. Cells co-expressing wildtype ANXA4-GFP and **k** Ca2Mut-RFP, **l** Ca4Mut-RFP, **m** TrimMut-RFP or **n** A4 ΔN-RFP were injured and fluorescence intensity measured at the site of injury. Results are representatives of at least three experiments for each condition. **o** Membrane integrity upon 55 °C heat shock in MCF7A4^{--}-CRISPR (#1) cells with introduced ANXA4-RFP or Ctrl-RFP and assayed by Hoechst-33258 exclusion as in **f**.

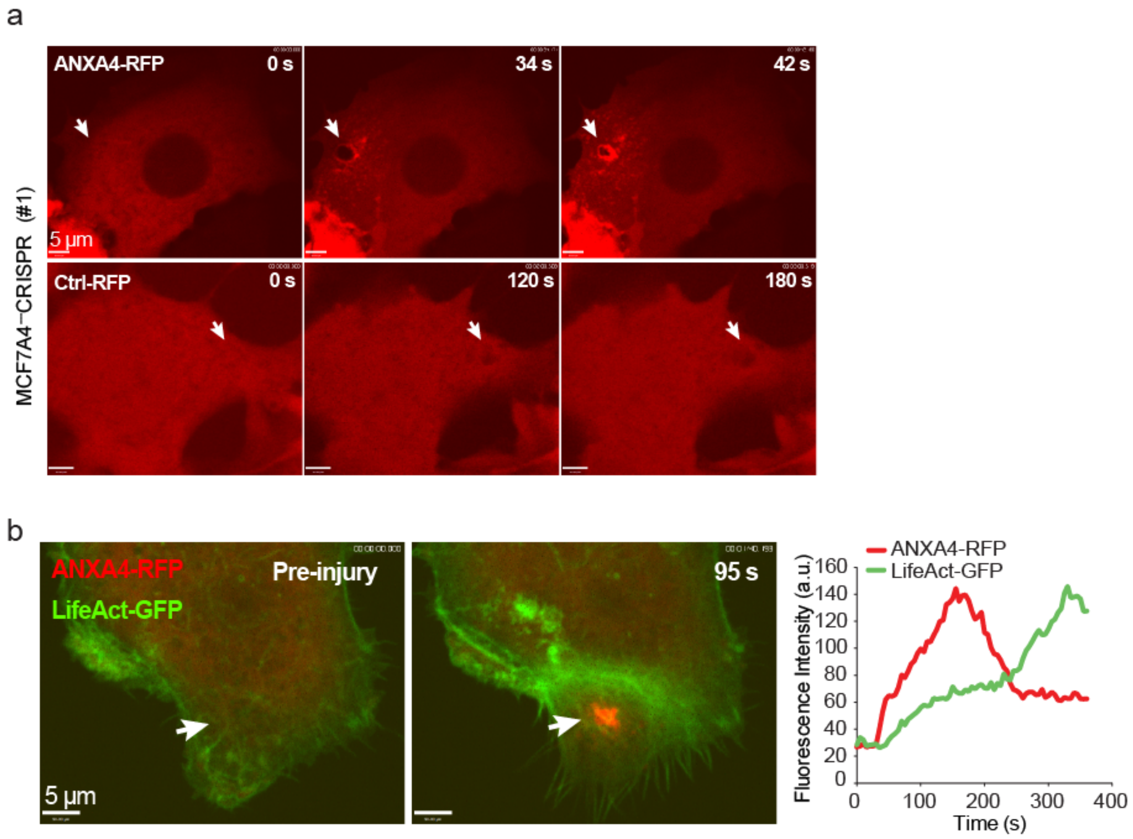

Supplementary Fig. 2 ANXA4 translocates to wound edges before actin accumulation. **a** 2D Images of MCF7A4^{--}-CRISPR cells before and after a large laser injury (arrow) showing localization of ANXA4-RFP to the wound edge and subsequent gradual wound closure as compared to Ctrl-RFP (lower panel). **b** Sequential images of ANXA4-RFP and LifeAct-GFP (F-actin marker) behavior in MCF7 cell before and after localized laser injury (white arrow) with corresponding fluorescence plot (right).

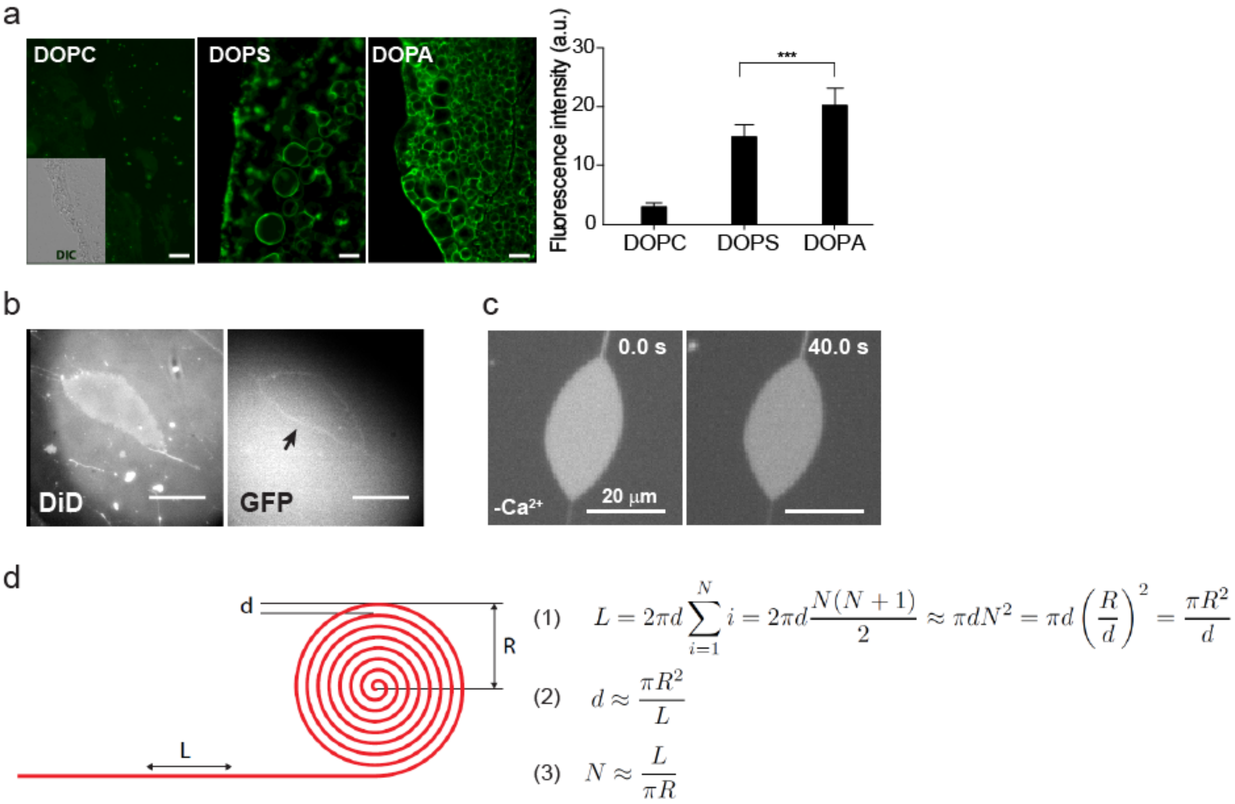

Supplementary Fig. 3 Effect of ANXA4 on membranes. **a** Representative images of giant liposomes with different lipid composition upon addition of recombinant ANXA4-GFP protein (50 µg/ml) in the presence of Ca^{2+} (2 mM). Phosphatidylcholine (DOPC) was used as carrier lipid constituting 90% of the liposomes, while phosphatidylserine (DOPS) or phosphatidic acid (DOPA) constituted 10%. 0.1% DOPE-Atto 647N and light microscopy was used to visualize the liposomes. Scale bar, 20 µm. Quantification (right plot) of ANXA4-GFP fluorescence intensity from 10 different areas with giant liposomes. *** $P \leq 0.001$ based on students t-test. **b** Membrane patch stained with DiD after addition of ANXA4-GFP protein showing initial binding of ANXA4-GFP to the edges of the membrane patch. **c** Membrane patch incubated with wtANXA4 in the absence of Ca^{2+}. **d** Model for rolling of planar membranes induced by binding of a curvature-inducing protein. A certain region (patch) of the membrane has initially a length L measured in the direction perpendicular to the rolling axis. After completion of rolling, a cylindrical roll with radius R has formed. The period length between layers inside the roll is d. The model is simplified by approximating the roll as concentric circles, which is a fair approximation when the number of turns N is large. Equations (1-3) provide relationships between N, d, R and L assuming that N>>1 and that the radius of the roll can be written as R=Nd.

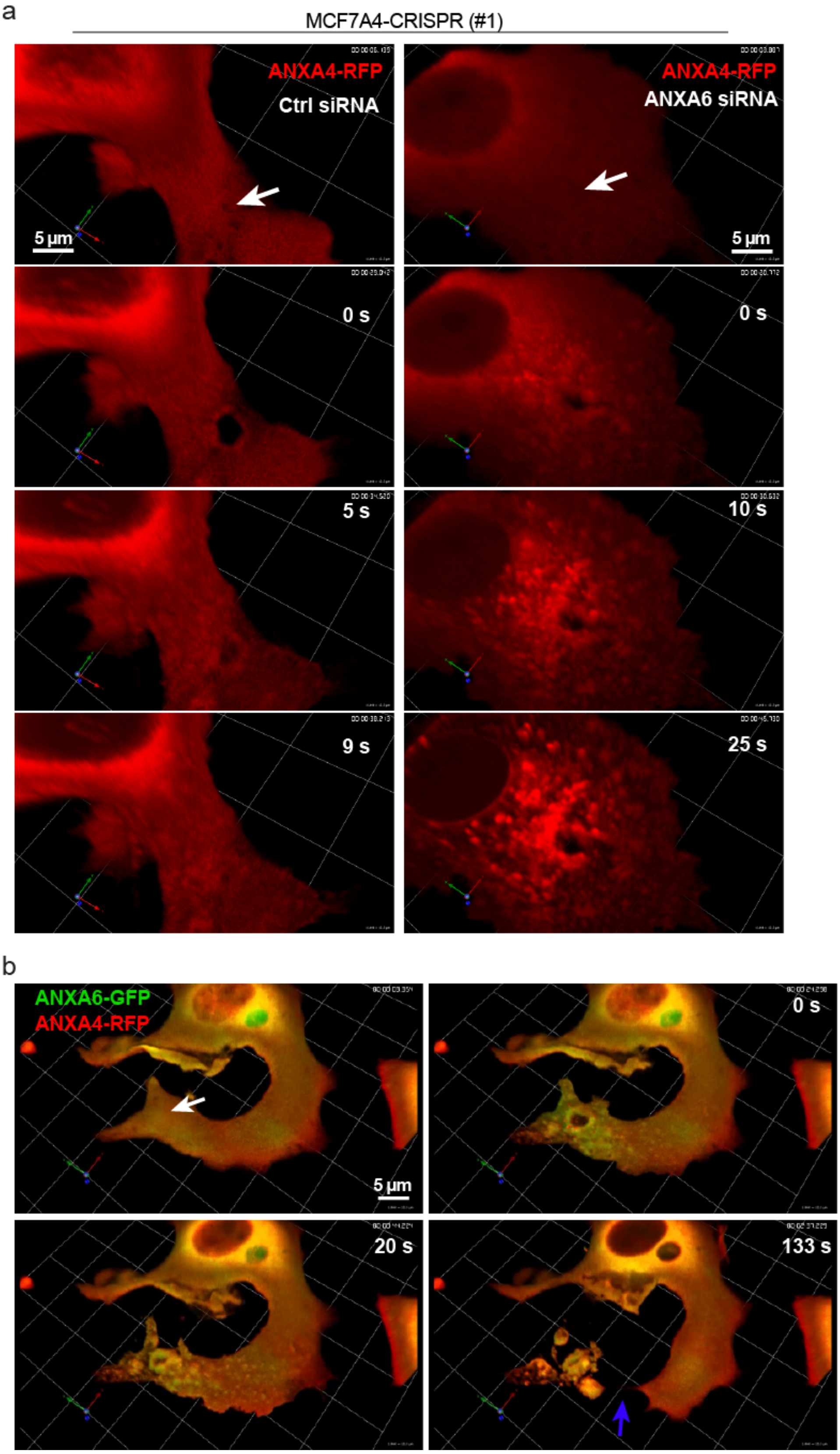

Supplementary Fig. 4 ANXA6 is needed for wound closure. **a** Sequential 3D images of MCF7A4^{--}-CRISPR cells expressing wildtype ANXA4-RFP, and treated with Ctrl or ANXA6 siRNA and exposed to laser injury (white arrow indicate injury site). Note: ANXA6 siRNA treated cell is eventually dying from the injury. **b** Representative sequential images of wound healing involving two mechanisms: first ANXA4-RFP/ANXA6-GFP translocation to wound edges initiates local wound closure. Secondly, excision of the damaged part of the membrane (blue arrow). Also see Supplementary Movie 3.

Supplementary note 1

Model for neck formation at a membrane hole

Definitions and assumptions

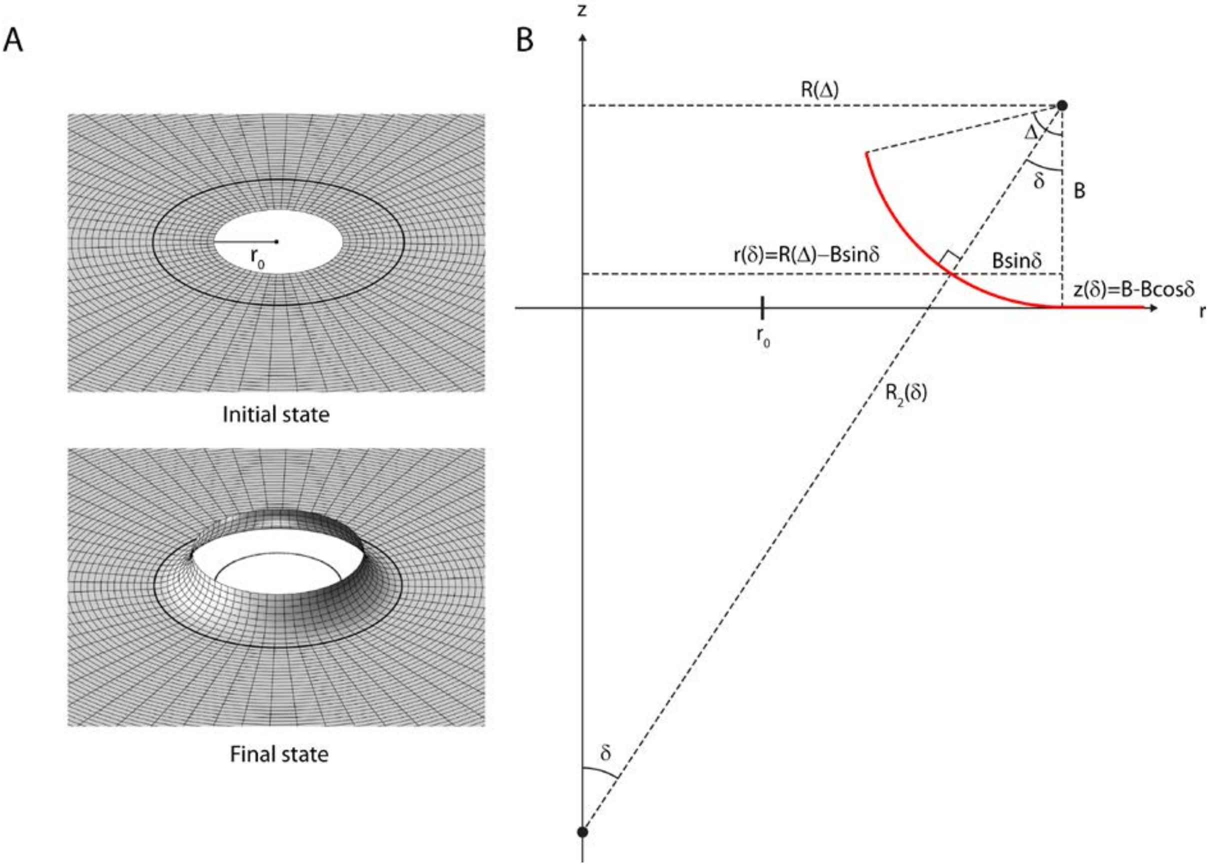

Supplementary Fig. 5 Schematic of the model for neck formation. **A** Initial and final (curved) state of the membrane hole. The area inside the solid circle contributes to the change in curvature elastic energy. **B** Profile of the neck and definitions of quantities used in the calculation.

We consider curvature close to a hole in a membrane with rotational axial symmetry. In the initial state, the membrane has a circular hole with radius r_0 and is planar. Upon binding of a protein (e.g. annexins) to the membrane surface the effect of the protein is modeled as the induction of a spontaneous curvature (c_0) of the combined membrane/protein sheet. This is assumed to result in a partial or complete rolling around the edge of the hole, leading to a neck-like geometry. The curvature radius of the roll is B and the rolling angle (denoted *neck angle*) is Δ. The circular profile of the neck is assumed based on the experimental results for membrane rolling in supported membrane patches. We also make the simplifying assumption that $B \cdot c_0 = 1$, i.e. that the in-plane radius of curvature is equal to the radius of spontaneous curvature. The difference in elastic energy $\Delta H(\Delta)$ between the final (curved) state and the initial state is calculated. The energetically favored final state corresponds to the rolling angle Δ_{min} that gives a minimum value of ΔH.

Description of model

The energy in the initial state is:

$$H_0 = H_{0\lambda} + H_{0A} \tag{1}$$

where $H_{0\lambda}$ is the energy contribution from line tension at the hole edge and H_{0A} is the

curvature elastic energy of the area A in the initial (planar) state.
The energy in the final state is:

$$H = H_\lambda + H_\mathrm{M} + H_\mathrm{G} \tag{2}$$

where H_λ is the energy contribution from line tension at the hole edge, H_M is the mean curvature elastic energy and H_G is the gaussian curvature elastic energy, both for the area A.
The total change in energy upon formation of the neck is:

$$\Delta H = H - H_0 = H_\lambda - H_{0\lambda} + H_\mathrm{M} + H_\mathrm{G} - H_{0A} \tag{3}$$

We therefore need to compute the terms in equation (3). The energy of the membrane edge is modeled by a line tension λ. The general expression for the curvature elastic energy H_curve of an area A is, according to Helfrich[1]:

$$H_\mathrm{curve} = H_\mathrm{M} + H_\mathrm{G} = \int_A \left[\frac{1}{2}k_c(\overline{c} - c_0)^2 + k_G\overline{c_G}\right] dA \tag{4}$$

where k_c is the *mean curvature elastic modulus* [J], k_G is the *gaussian curvature elastic modulus* [J] and c_0 is the spontaneous curvature $[m^{-1}]$. The local curvature of the membrane is described by the two principal radii of curvature, R_1 and R_2. The mean curvature is defined as: $\overline{c} = \frac{1}{R_1} + \frac{1}{R_2}$ and the gaussian curvature as: $\overline{c_G} = \frac{1}{R_1}\frac{1}{R_2}$.

Area A of the neck region

The area A of the curved neck region is marked by the black circle in Supplementary Figure 5A. As we only consider bending deformations in this model, A must remain constant between the initial and the final states, so that the energy contribution from an area change is zero. This requirement determines the value of $R(\Delta)$, that is the r-coordinate of the center of the circle defining the in-plane curvature. The area in the initial (flat) state is:

$$A_\mathrm{initial} = \pi R^2 - \pi r_0^2 \tag{5}$$

The area in the final (curved) state is:

$$\begin{aligned} A_\mathrm{final} = \int_A d\,A = \int_0^\Delta 2\,\pi r B d\delta = \int_0^\Delta 2\,\pi(R - B\sin\delta)Bd\delta \\ = 2\pi B(R\Delta + B\cos\Delta - B) \end{aligned} \tag{6}$$

The requirement $A_\mathrm{final} = A_\mathrm{initial}$ gives the following expression for R:

$$\frac{R}{B} = \Delta + \sqrt{\Delta^2 + (\frac{r_0}{B})^2 + 2(\cos\Delta - 1)} \tag{7}$$

or when $B \cdot c_0 = 1$:

$$Rc_0 = \Delta + \sqrt{\Delta^2 + (r_0 c_0)^2 + 2(\cos\Delta - 1)} \tag{8}$$

Radii of curvature R_1, R_2

In order to compute the mean and gaussian curvature elastic energy in the final state we need to know the radii of curvature R_1 and R_2 at any point on the curved neck. Referring to Supplementary Figure 5B we define the in-plane radius of curvature as the constant $R_1 = B$. The out-of-plane radius of curvature is found according to the lower triangle in Supplementary Figure 5B:

$$\frac{1}{R_2} = -\frac{\sin\delta}{r(\delta)} \tag{9}$$

The angle $\delta \in [0, \Delta]$ describes any point on the cylindrically symmetric neck and $r(\delta) = R - B\sin\delta$. The minus in equation (9) is due to the opposite curvature with respect to R_1. Finally, we get for the mean and gaussian curvature:

$$\bar{c} = \frac{1}{R_1} + \frac{1}{R_2} = \frac{1}{B} - \frac{\sin\delta}{r} \qquad \overline{c_G} = \frac{1}{R_1}\frac{1}{R_2} = -\frac{1}{B}\frac{\sin\delta}{r} \tag{10}$$

Change in line tension energy: $\boldsymbol{H_\lambda - H_{0\lambda}}$

The line tension energy increases due to expansion of the hole. The change is:

$$H_\lambda - H_{0\lambda} = 2\pi\lambda r(\Delta) - 2\pi\lambda r_0 \tag{11}$$

$$= 2\pi\lambda B(\frac{R}{B} - \frac{r_0}{B} - \sin\Delta) \tag{12}$$

$$= 2\pi\frac{\lambda}{c_0}(Rc_0 - r_0c_0 - \sin\Delta) \tag{13}$$

where λ is the line tension for a free membrane edge. Equation (13) is valid for $B \cdot c_0 = 1$.

Elastic bending energy in the initial state: $\boldsymbol{H_{0A}}$

In the initial state, the membrane is flat and $R_1 = R_2 = \infty$. According to equation (4), the elastic bending energy in the initial state becomes:

$$H_{0A} = \frac{1}{2}k_c c_0^2 A \tag{14}$$

Inserting the area from equation (5) gives:

$$H_{0A} = \frac{\pi}{2}k_c c_0^2(R^2 - r_0^2) \tag{15}$$

Gaussian curvature elastic energy in the final state: $\boldsymbol{H_G}$

The gaussian curvature elastic energy in the final state is:

$$H_G = \int_A k_G\, \overline{c_G} dA \tag{16}$$

$$= \int_A k_G \frac{1}{R_1}\frac{1}{R_2} dA \tag{17}$$

$$= -\int_0^\Delta k_G \frac{1}{B}\frac{\sin\delta}{r} 2\pi r B d\delta \tag{18}$$

$$= 2\pi k_G(\cos\Delta - 1) \tag{19}$$

Note that this result is independent of r_0 and c_0.

Mean curvature elastic energy in the final state: $\boldsymbol{H_M}$

The mean curvature elastic energy in the final state is:

$$H_M = \int_A \frac{1}{2}k_c(\bar{c} - c_0)^2 dA \tag{20}$$

$$= \int_A \frac{1}{2}k_c(\frac{1}{B} - \frac{\sin\delta}{r} - c_0)^2 2\pi r B d\delta \tag{21}$$

With the assumption $B \cdot c_0 = 1$ this becomes:

$$H_M = \pi k_c \int_0^\Delta \frac{\sin^2\delta}{rc_0} d\delta \tag{22}$$

$$= \pi k_c \int_0^\Delta \frac{\sin^2\delta}{Rc_0 - \sin\delta} d\delta \tag{23}$$

The integral in equation (23) is evaluated numerically for practical reasons.

Supplementary note 2

Results

To evaluate equation (3) we need values for the parameters: λ, k_c, k_G and c_0. Precise values corresponding to our experimental system (POPC, POPS 9:1 or cellular membranes) are not available in the literature and we therefore use the following order-of-magnitude estimates based on similar lipid systems:

λ= 10 pN. Reference [2] reports the values 6.9 pN and 20.7 pN for DOPC from the companies Sigma and Avanti respectively.
k_c=4.0$\cdot\ 10^{-20}$ J. From Marsh[3], table II.10.4.1, page 474. Reported values for POPC are from 2.5-8.5$\cdot\ 10^{-20}$ J.
k_G=-3.0$\cdot\ 10^{-20}$ J. From Marsh[3], table II.10.4.2, page 477. Based on monolayer results showing $k_G/k_c \simeq -0.75$
c_0=0.033 nm^{-1}. Based on computational modeling of Shiga toxin by Pezeshkian et. al. [4].

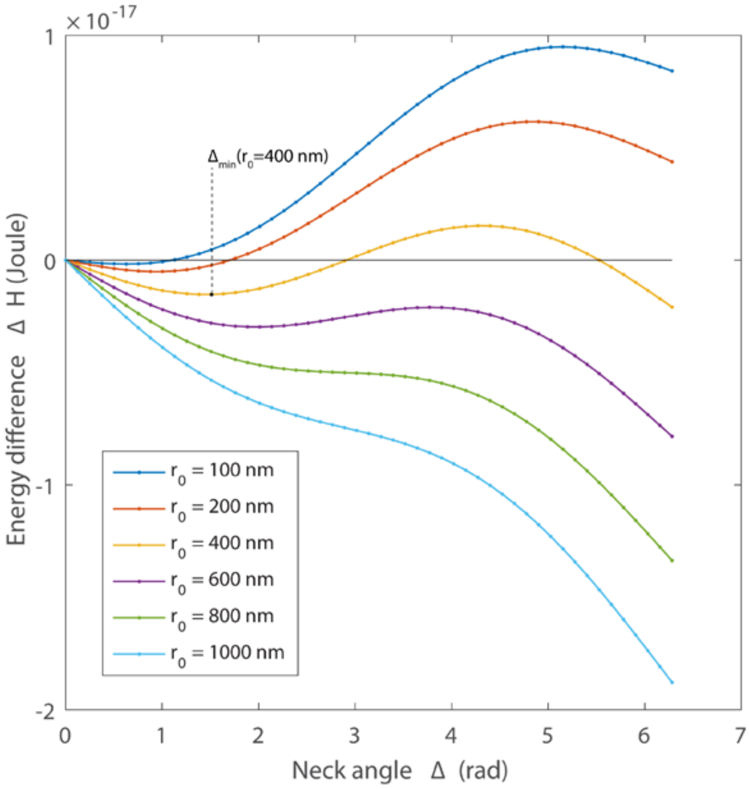

Supplementary Fig. 6 Energy difference ΔH in equation (3) as a function of the neck angle Δ for varying values of the initial hole size r_0. For values of r_0 below a critical hole radius r_0* there is a minimum in ΔH corresponding to an energetically favored neck angle Δ_{min}. In the plot $r_0^* \approx$ 800 nm. For $r_0 > r_0^*$, ΔH decreases monotonically with Δ meaning that rolling is favored.

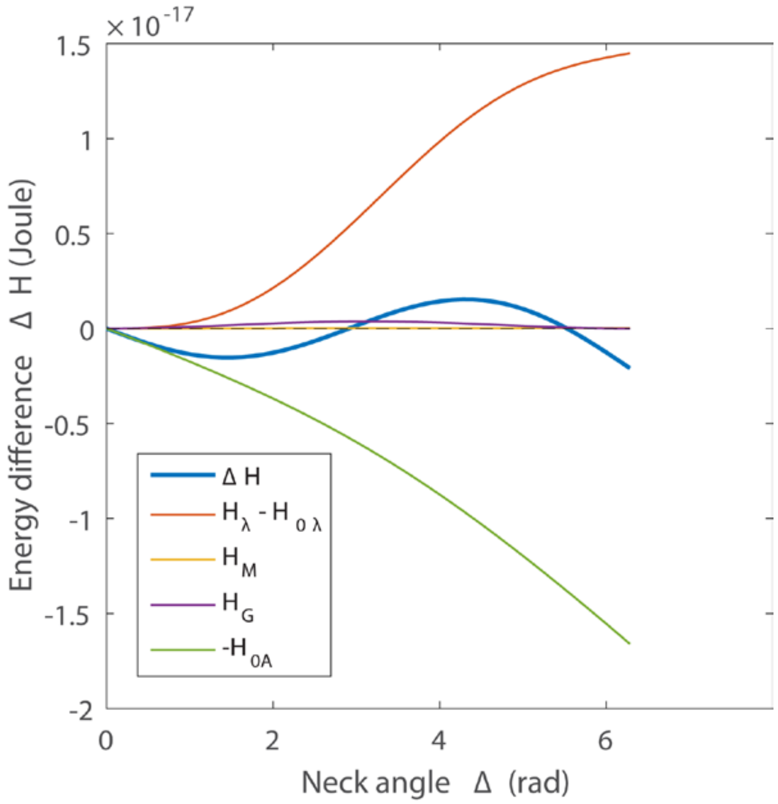

Supplementary Fig. 7 Contributions to the energy change ΔH from the terms in equation (3) for r_0 = 400 nm. Note that the increase in line tension energy (H_λ-$H_{0\lambda}$) and the curvature energy in the initial state (-H_{0A}) dominates.

To obtain an approximate expression for the critical hole radius r_0^* we neglect the terms (H_M) and (H_G) in equation (3) and expand in Δ. This gives:

$$\Delta H \simeq 2\pi \frac{\lambda}{c_0}(\Delta - \sin\Delta) - \pi k_c r_0 c_0 \Delta \tag{24}$$

We find Δ_{min} by differentiation:

$$\left.\frac{\partial \Delta H}{\partial \Delta}\right|_{\Delta_{min}} = 0 \tag{25}$$

$$\Delta_{min} \simeq \cos^{-1}\left[1 - \frac{k_c c_0^2 r_0}{2\lambda}\right] \tag{26}$$

This has a solution only if $r_0>0$ and if:

$$r_0 < \frac{4\lambda}{k_c c_0^2} \simeq r_0^* \tag{27}$$

-providing an approximate value of the critical hole radius r_0^*. Equation (26) is in Supplementary Fig. 8 compared to the corresponding value determined numerically.

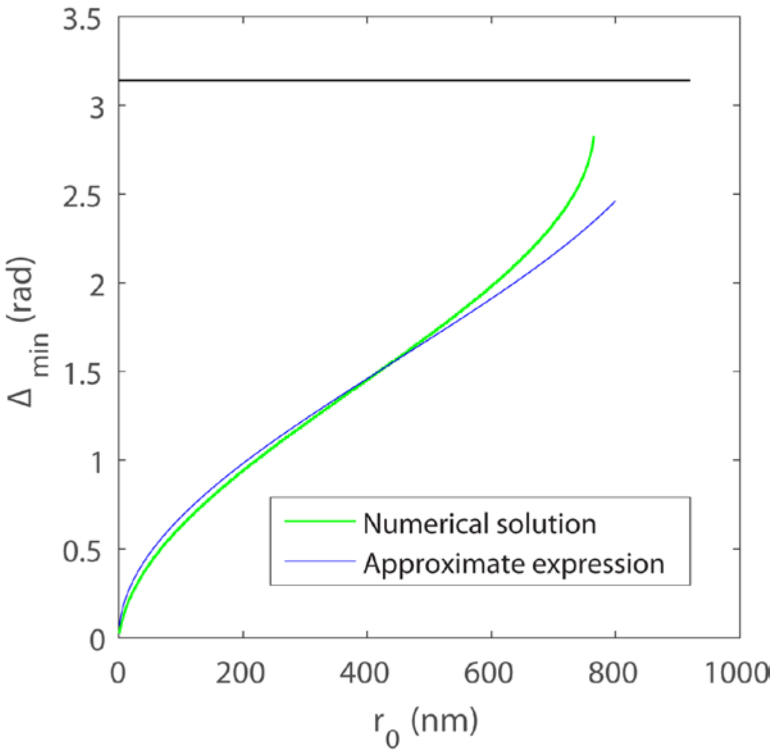

Supplementary Fig. 8 Variation in the energetically favored neck angle $\boldsymbol{\Delta_{min}}$ with initial hole radius $\boldsymbol{r_0}$. Green line is determined numerically from equation (3). Blue line is the approximate solution from equation (26).

References

[1] Helfrich, W. Elastic properties of lipid bilayers: theory and possible experiments. Z. Naturforsch. 28, 693-703 (1973).
[2] Karatekin, E. et al. Cascades of transient pores in giant vesicles: line tension and transport. Biophys. J. 84, 1734-1749 (2003).
[3] Marsh, D. Handbook of lipid bilayers (CRC press, 2013).
[4] Pezeshkian, W. et al. Membrane invagination induced by shiga toxin B-subunit: from molecular structure to tube formation. Soft Matter 12, 5164-5171 (2016).
