# Supplementary material for: Annexin A4 and A6 induce membrane curvature and constriction during cell membrane repair
Source: Nat Commun. 2017 Nov 20;8:1623. doi: 10.1038/s41467-017-01743-6 (PMC5696365; doi:10.1038/s41467-017-01743-6)
Supplement: Supplementary file 2 — Description of Additional Supplementary Files [file 41467_2017_1743_MOESM2_ESM.pdf]

**Description of Additional Supplementary Files**

File Name: Supplementary Movie 1
Description: **ANXA4 induces membrane conformation and wound closure in MCF7 cells** (Related to Fig. 2). 3D Movie showing ANXA4-RFP (first clip) and A4 TrimMut-RFP (second clip) localization upon laser injury in MCF7A4$^{--}$-CRISPR cells. Scale: 1 unit = 12.3 μm.

File Name: Supplementary Movie 2
Description: **ANXA4 triggers membrane curvature and rolling of supported membrane patches** (Related to Fig. 3). Supported secondary membrane patches (90% POPC, 10% POPS. DiD stain) before and after addition of recombinant ANXA4-GFP protein or A4 TrimMut protein in the presence of $Ca^{2+}$. Scale bar, 50 μm.

File Name: Supplementary Movie 3
Description: **Wound healing implicating two distinct mechanisms** (Related to Fig. 6 and Supplementary Fig. 4b). First, ANXA4-RFP/ANXA6-GFP translocation to wound edges and initiation of local wound closure. Secondly, excision of the damaged part of the membrane including the hole. Scale: 1 unit = 12.3 μm.
